# HDAC Inhibitors Increase NRF2-Signaling in Tumour Cells and Blunt the Efficacy of Co-Adminstered Cytotoxic Agents

**DOI:** 10.1371/journal.pone.0114055

**Published:** 2014-11-26

**Authors:** Michael McMahon, Kathryn H. Campbell, A. Kenneth MacLeod, Lesley A. McLaughlin, Colin J. Henderson, C. Roland Wolf

**Affiliations:** Medical Research Institute, University of Dundee, Dundee, United Kingdom; Taipei Medicine University, Taiwan

## Abstract

The NRF2 signalling cascade provides a primary response against electrophilic chemicals and oxidative stress. The activation of NRF2-signaling is anticipated to have adverse clinical consequences; NRF2 is activated in a number of cancers and, additionally, its pharmacological activation by one compound can reduce the toxicity or efficiency of a second agent administered concomitantly. In this work, we have analysed systematically the ability of 152 research, pre-clinical or clinically used drugs to induce an NRF2 response using the MCF7-AREc32 NRF2 reporter. Ten percent of the tested drugs induced an NRF2 response. The NRF2 activators were not restricted to classical cytotoxic alkylating agents but also included a number of emerging anticancer drugs, including an IGF1-R inhibitor (NVP-AEW541), a PIM-1 kinase inhibitor (Pim1 inhibitor 2), a PLK1 inhibitor (BI 2536) and most strikingly seven of nine tested HDAC inhibitors. These findings were further confirmed by demonstrating NRF2-dependent induction of endogenous *AKR* genes, biomarkers of NRF2 activity. The ability of HDAC inhibitors to stimulate NRF2-signalling did not diminish their own potency as antitumour agents. However, when used to pre-treat cells, they did reduce the efficacy of acrolein. Taken together, our data suggest that the ability of drugs to stimulate NRF2 activity is common and should be investigated as part of the drug-development process.

## Introduction

NF-E2 p45-related factor 2 (Nrf2), a cap ‘n’ collar (CNC) basic-region leucine zipper (bZIP) transcription factor regulates a transcriptional programme that enables cells to withstand transient periods of exposure to stress [1]. This evolutionarily-conserved transcriptional programme involves the binding of NRF2 to the Antioxidant Response Element (ARE), a DNA element found in the promoters of numerous genes involved in drug detoxication (glutathione *S*-transferases, aldo-keto reductases), drug transport and anti-oxidant defense, such as haem oxygenase 1, malic enzyme, thioredoxin and glucose 6-phosphate dehydrogenase. Moreover, NRF2 can enhance the activity of key pathways involved in maintaining proteostasis, including the 26S proteasome and autophagy [2]. These adaptations and others collectively confer a survival phenotype upon cells that minimises damage to their functional and structural integrity.

Under normal metabolic conditions, NRF2 activity is restrained by the CRL3^KEAP1/KEAP1^ ubiquitin ligase complex [3]. Kelch-like ECH-associated Protein 1 (KEAP1) provides a substrate recognition signal for the Cullin-3-Rbx1 Ligase (CRL3) holoenzyme leading to the transcription factor’s rapid ubiquitylation and degradation. Normally, it is only in stressed cells that NRF2 transiently accumulates and initiates an adaptive response. This accumulation results from the inactivation of KEAP1 by ‘danger’ signals, such as zinc or lipid peroxidation products, or toxic electrophiles [4].

The activation of the NRF2 signalling cascade is an adaptive response which generates resistance to further cytotoxic stress, resulting in cell survival [5]. While transient activation of NRF2 in normal cells is desirable, persistent activation of NRF2 is deleterious [6]. For example, in the setting of cancer, recurrent mutations in KEAP1 and NRF2 resulting in its constitutive activation have been observed in up to 34% of lung tumours [7]–[9] and also in several other types of tumours, including head-and-neck, skin, prostate, and pancreatic cancers [7],[10]. Constitutive NRF2 signalling is believed to benefit the neoplastic cells (and is thus detrimental to the host organism) because it facilitates cell proliferation (10). Also, NRF2 activation will boost cell survival and prevent apoptosis [12]. For similar reasons, it may also play a role in chemo-resistance [2]. Related to these effects, mutations in KEAP1 and NRF2 are associated with a poor prognosis in lung cancer patients [11]. Uncontrolled activation of NRF2 might also be harmful in the context of normal cells as genetic or pharmacological activation of NRF2 causes epidermal thickening and hyperkeratosis in mice that resemble the human disease lamellar ichythosis [13].

These examples – and the fact that NRF2 activity is so tightly controlled – suggest that opportunistic stimulation of NRF2 signalling by drugs used in the treatment of cancer is undesirable. Not only will it potentially contribute to the survival and proliferation of pre-malignant cells, it may also give rise to unexpected drug-drug interactions as a consequence of NRF2’s ability to induce drug detoxication genes. In this study, we set out to examine systematically the frequency with which clinical drugs or drugs in development can activate NRF2 signalling. Our data suggest that this ability is a common feature of a small but significant fraction of therapeutic agents – including in particular histone deacetylase (HDAC) inhibitors.

## Materials and Methods

### Cell line

The MCF7-AREc32 cell line was derived in the authors’ laboratory and has been previously described [14]. The growth medium was DMEM with glutamax, supplemented with 10% (v/v) fetal bovine serum, 1x penicillin-streptomycin supplement, and 0.8 mg/ml G418. A-431 (ATCC CRL-1555) cells were obtained from ATCC and cultured in DMEM with glutamax, supplemented with 10% (v/v) fetal bovine serum, 1x penicillin-streptomycin. Cells were maintained at 37°C in a humidified atmosphere containing 5% CO_2_.

### Chemicals

Chemicals were from commercial suppliers or academic collaborators. We provide a full description of each chemical, including name, source, putative therapeutic target and screening clinical status in Table S1. Compounds were dissolved in dimethyl sulfoxide at a final concentration of 10 mM (or at maximum solubility), and stored at −20°C.

### Luciferase activity assay

Luciferase activity was measured using the Luciferase Assay System (Promega), according to the manufacturer’s instructions. Briefly, cells in each well of a 96-well plate were washed with PBS and lysed in 30 µl of the lysis buffer provided with the kit. A 5 µl portion of the lysate was mixed with 25 µl of Luciferase assay reagent and the luminescence was quantified using the Orion II Microplate Luminometer (Berthold Detection Systems).

### Cell viability assay

The Adenosine 5′-triphosphate (ATP) bioluminescent somatic cell assay kit (Sigma-Aldrich) was used to determine cell viability, as described by the manufacturer. Briefly, cells in each well of a 96-well plate were lysed in 45 µl of the Somatic Cell ATP Releasing Reagent. Of this volume of lysate, 30 µl was transferred to a white-walled 96-well plate. To this was added 30 µl of resuspended Adenosine 5′triphosphate (ATP) Assay Mix and the resulting luminescence measured using the Orion II Microplate Luminometer (Berthold Detection Systems). To calculate Relative IC_50_ values, the resulting data were fitted to a Sigmoidal Dose-Response Curve (variable slope), as implemented in GraphPad Prism. The TOP parameter was fixed at 100% cell viability.

### Screen of MCF7-AREc32 cell line

The screen was performed in two stages. An initial primary screen was performed during which MCF7-AREc32 cells were seeded into 96-well plates at 5000 cells/well. After 24 h, single wells of cells were exposed to a 7-point ten-fold serial dilution series of each compound starting at 50 µM. After a further 24 h had elapsed, the luciferase activity and cell viability in control and treated cells was determined. Only those chemicals that displayed at least a two-fold increase in Luciferase activity at minimally one dose were taken forward into the secondary screen. For the secondary screen, the MCF7-AREc32 cells were plated as for the primary screen. They were treated in triplicate with an 11-point, four-fold dilution series of each test compound. Luciferase activity, and, using a separate set of plates, cell viability, was assayed at the 24 h time point. For each chemical, we determined the fold-change in Luciferase activity compared with control wells (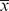 ± S.E.M, n = 3). Compounds that caused a fold increase in Luciferase activity that was significantly greater than two (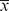 –2*S.E.M>2) while causing no greater than a 20% decline in cell viability were considered to stimulate NRF2 activity.

### Immunoblots

Whole-cell lysates were prepared by scraping cells into ice-cold radioimmune precipitation assay buffer (50 mM Tris-Cl, pH 7.4, 150 mM NaCl, 1% (v/v) Nonidet P-40, 0.5% (w/v) deoxycholic acid, 0.1% (w/v) SDS). Lysates were clarified by centrifugation (16,000 × *g*, 15 min, 4°C). Protein determination, SDS/polyacrylamide-gel electrophoresis and immunoblotting were carried out as previously described [15]. Antibodies used included rabbit sera raised against AKR1B10 [16], AKR1C1 [17], NRF2 [18] a mouse monoclonal antibody raised against AKR1C3 (gift from Prof. Trevor Penning, University of Pennsylvania) and a mouse anti-GAPDH antibody, clone GAPDH_71.1 (Sigma).

### Relative quantification of mRNA species

This was carried out by TaqMan chemistry. Total RNA was isolated from MCF7-AREc32 cells using Ambion RNA Extraction Kit (Ambion) followed by DNase treatment (RQ1 RNase-free DNase, Promega). Approximately 0.4 µg of total RNA was reverse-transcribed to cDNA using random hexamers (Promega) and the ImProm II Reverse Transcription System (Promega) according to the manufacturer’s instructions. The PCR mixes were prepared by mixing 6 µl of cDNA (diluted 1∶40) with 0.75 µl of TaqMan probe set and 7.5 µl of Master Mix (PerkinElmer Applied Biosystems). For the real-time PCR analysis, the following pre-designed TaqMan probe sets in solution were used: *AKR1C1*: Hs00413886; *Luciferase*: Mr03987587; 18 s ribosomal RNA: Hs03003631 (all from PerkinElmer Applied Biosystems). Data acquisition and analysis utilised the ABI PRISM 7700 sequence detection system (PerkinElmer Applied Biosystems). The relative gene expression levels in different samples were calculated using the Comparative C_T_ Method as outlined in the ABI PRISM 7700 Sequence Detection System User Bulletin #2. The expression of 18 s rRNA was used as the internal control.

### NRF2 knock-down

1.5×10^6^ MCF7-AREc32 cells were reverse transfected in 100-mm Tissue Culture dishes with 40 µl LipofectAMINE RNAiMAX (Invitrogen) and 250 pmoles of siRNA. The siRNAs that were used were ON-TARGETplus Non-targeting siRNA #4 (Dharmacon) and ON-TARGET plus SMARTpool human NFE2L2 (Dharmacon).

## Results

### Drug-induced NRF2-ARE signalling

**Figure 1:**
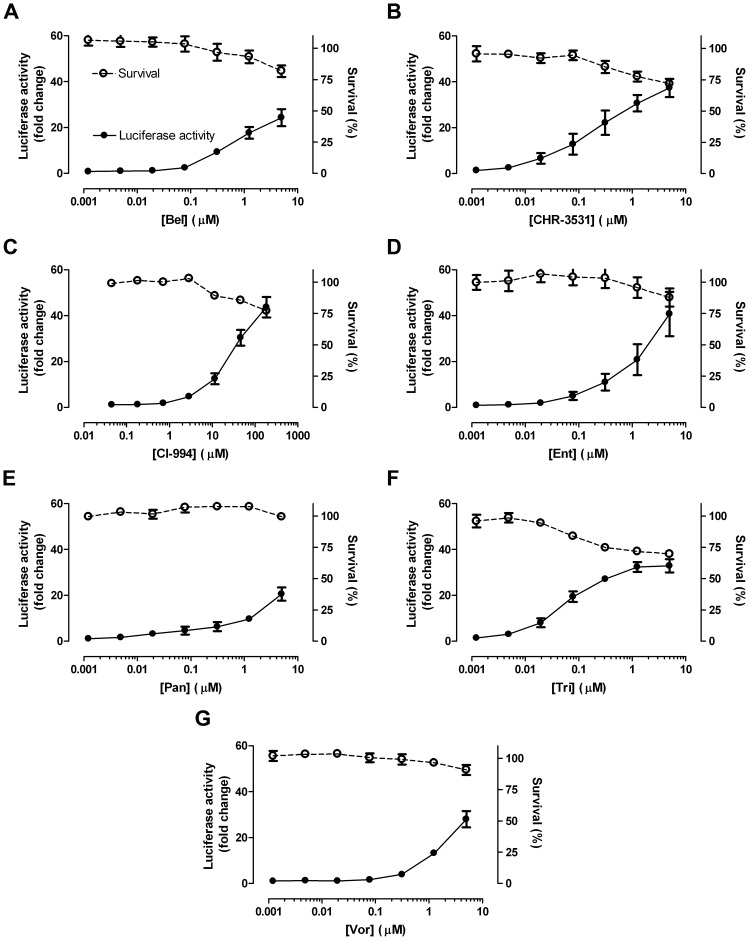
HDAC inhibitors stimulate ARE activity. Cell viability and luciferase activities were measured in separate plates of MCF7-AREc32 cells exposed to the indicated doses of the noted HDAC inhibitors for 24 h. Each measured parameter is plotted as 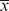 ±S.E.M of three independent experiments.

As previously described, the MCF7-AREc32 cell line contains low levels of NRF2 and, therefore, low levels of luciferase expression [14]. This makes it an excellent cell model with which to identify drugs that activate NRF2 signalling. We assembled a library of 152 drugs which target a variety of biochemical pathways (Table S1); the selection of compounds, however, was made with no fore-knowledge or preconception of their likelihood to stimulate NRF2 signalling. An initial primary screen using this library identified 46 candidate chemicals that elevated luciferase activity in the MCF7-AREc32 cell line (Table S1). The more extensive secondary 11-point cytotoxicity screen confirmed that thirteen of these compounds induced ARE-driven luciferase expression (Figs. S1 & S2 and Fig. 1). Surprisingly, only three of the thirteen activators (carmustine, lomustine, melphalan) are conventional cytotoxic drugs. The remainder of the active compounds were newer targeted therapies and they included a PLK1 inhibitor (BI 2536), a PIM-1 kinase inhibitor (Pim-1 inhibitor 2), and an IGF-1R inhibitor (NVP-AEW541). Most strikingly, however, we observed that seven of nine tested HDAC inhibitors (belinostat (Bel), CHR-3531, CI-994, entinostat (Ent), panobinostat (Pan), trichostatin A (Tri) and vorinostat (Vor)) increased Luciferase activity. Besides these thirteen activators, our screen identified two inhibitors of NRF2-ARE signalling: Epirubicin, a DNA-intercalating agent, and Bexarotene, an RXR agonist (Fig. S3). Collectively, these data showed that approximately 10% of the compounds tested altered NRF2-ARE signalling.

### PIM-1 kinase and IGF-1R inhibitors induced ARE activity

**Figure 2:**
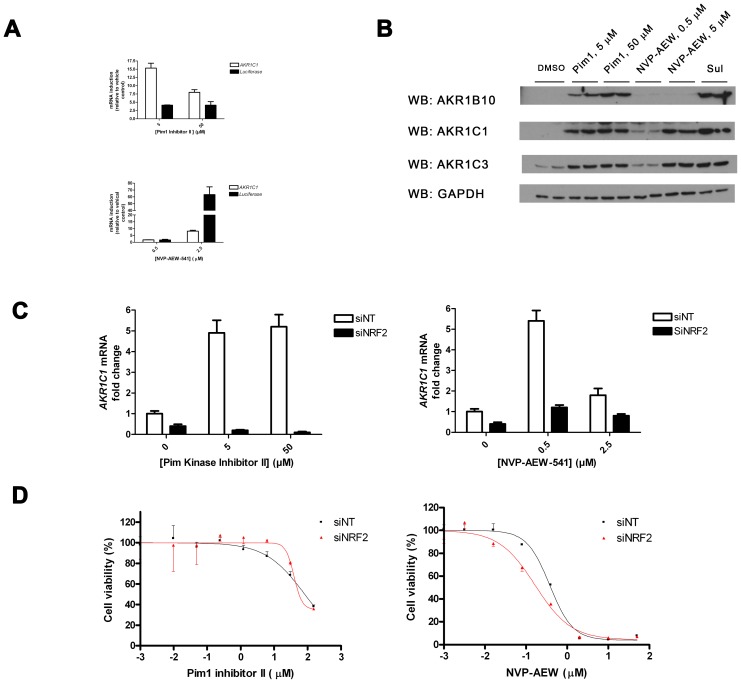
Pim 1 inhibitor II and NVP-AEW increase ARE activity. **A & B**, MCF7-AREc32 cells were exposed to the indicated doses of Pim1 inhibitor 2 (Pim1), NVP-AEW541 or SFN. After 24 h had elapsed, total RNA was prepared, reverse-transcribed to cDNA and levels of *AKR1C1* and *luciferase* mRNA was measured by real-time PCR (A). Alternatively, whole-cell lysates were prepared from duplicate dishes of cells and blotted for the indicated proteins (B). GAPDH serves as a loading control. **C,** MCF7-AREc32 cells were treated with non-targeting (siNT) or NRF2 targeting (siNRF2) siRNAs and, 48 h later, they were exposed to different doses of the indicated chemicals. After 24 h had elapsed, mRNA was prepared, and the amount of *AKR1C1* mRNA determined by real-time quantitative PCR **D,** MCF7-AREc32 cells were treated with non-targeting (siNT) or NRF2 targeting (siNRF2) siRNAs and, 48 h later, they were exposed to different doses of the indicated chemicals. Cell viability was assessed 72 h later. Data are presented as 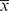 ±S.E.M of three independent experiments.

We confirmed by TaqMan mRNA analysis and Western-blot analysis that Pim1 inhibitor 2 and NVP-AEW541 stimulated expression not only of the luciferase reporter but also of previously authenticated NRF2-target genes. For example, both compounds increased the steady-state level of various members of the Aldo-Keto Reductase (AKR) family of proteins [16] (Fig. 2A & B). Moreover, we successfully knocked-down NRF2 mRNA (Fig. S4A) and protein (Fig. S4B) using RNAi; this approach was used to reveal that the enhanced AKR expression in response to both Pim1 inhibitor 2 and NVP-AEW541 was mediated at least in part via NRF2 signalling (Fig. 2C). Sulforaphane (SFN), the prototypic NRF2 inducer, was included in these experiments as a positive control [19]. As expected, the ability of SFN to boost AKR expression showed absolute dependency on NRF2; this result suggests that both NVP-AEW541 and Pim1 inhibitor 2 may additionally influence AKR expression by means other than NRF2 (Fig. 2C). Finally, we found that the cytotoxicity of NVP-AEW541 (but not Pim1 inhibitor 2) was modestly impaired by NRF2 signalling, as knock-down of NRF2 significantly (P<0.05, compared with non-template control cells) reduced the chemical’s Relative IC_50_ value in this cell line (Fig. 2D).

### HDAC inhibitors increase expression of ARE-activated genes

**Figure 3:**
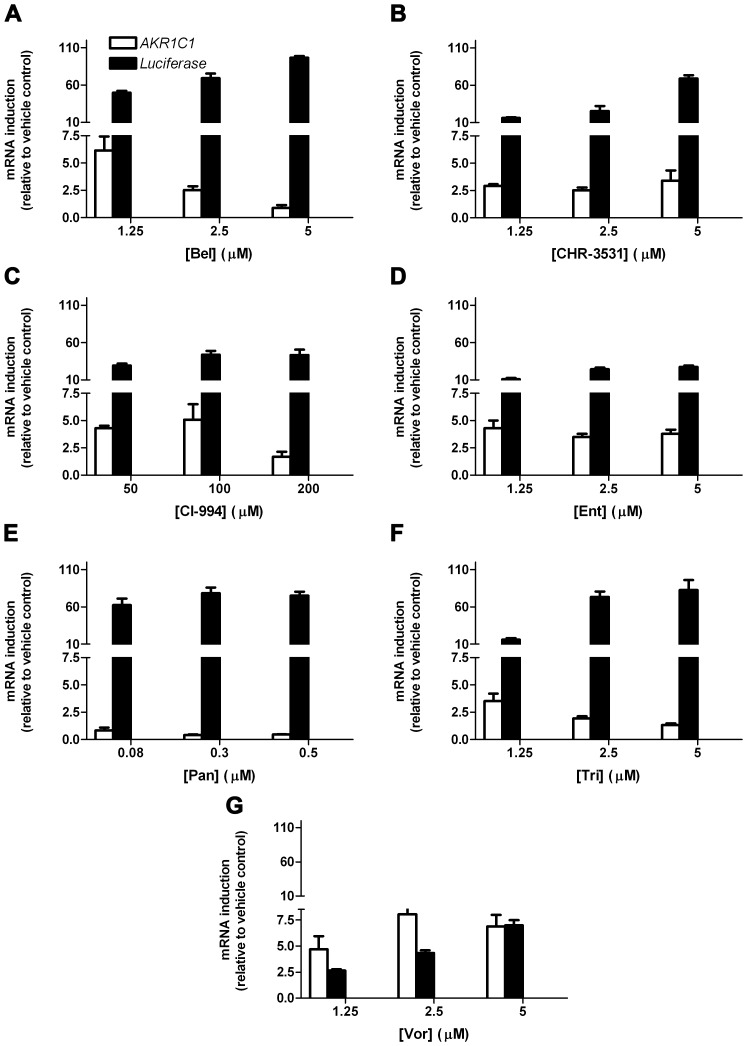
HDAC inhibitors increase expression of *AKR1C1* mRNA. **A – G**, MCF7-AREc32 cells were cultured for 24 h in the presence of the indicated concentrations of the stated HDAC inhibitors. The expression levels of *AKR1C1* and *Luciferase* mRNAs (relative to vehicle control) was determined by real-time quantitative PCR. Data are plotted as 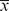 ±S.E.M of three independent experiments.

**Figure 4:**
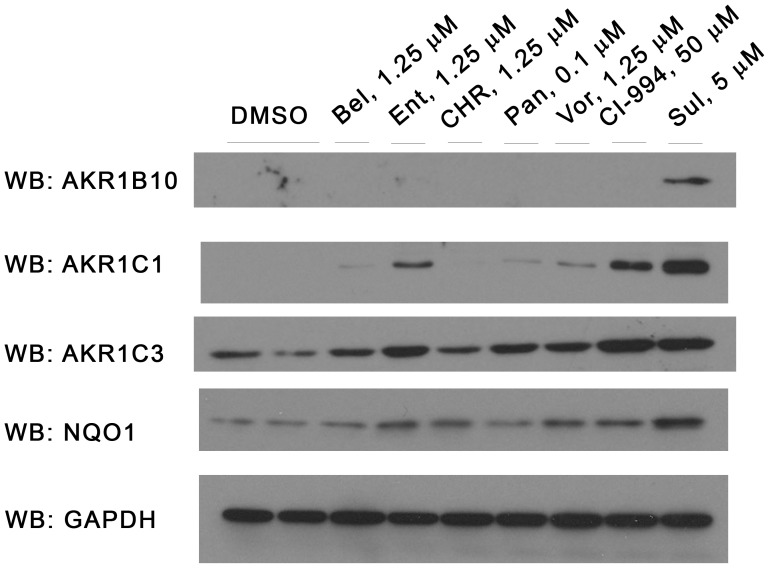
HDAC inhibitors increase expression of a range of AKR proteins. MCF7-AREc32 cells were treated with the stated concentration of chemicals for 24 h. Whole-cell lysates were prepared and blotted for the indicated proteins.

Quantitative RT-PCR confirmed that six of seven separate HDAC inhibitors upregulated mRNA encoding not only *luciferase* but also the endogenous NRF2-regulated *AKR1C1* gene (Fig. 3A – G). The exception was Pan (Fig. 3E). In general, these drugs had a considerably more profound effect on *luciferase* expression than on *AKR1C1* expression. Vor was an outlier to this trend; it displayed more pronounced activation of *AKR1C1* than it did *luciferase* (Fig. 3G). Upregulation of *AKR1C1* mRNA was paralleled by an increase in the corresponding protein (Fig. 4).

**Figure 5:**
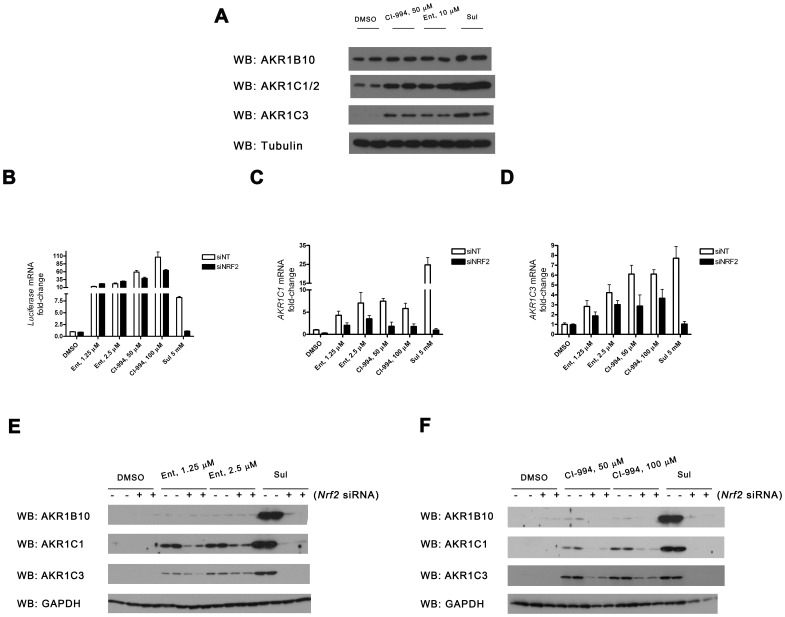
HDAC inhibitors increase ARE-driven gene expression in part via NRF2. **A,** Duplicate dishes of A-431 cells were treated with CI-994, Ent or vehicle control for 24 h and blotted for the indicated proteins. **B – F,** MCF7-AREc32 cells were treated with non-targeting (siNT) or NRF2 targeting (siNRF2) siRNAs and, 48 h later, they were exposed to different doses of the indicated chemicals for 24 h. The expression level of *Luciferase* (B), *AKR1C1* (C) or *AKR1C3* (D) was determined by real-time quantitative PCR. Protein levels (E & F) were determined by immunoblot. Data are presented as 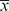 ±S.E.M of three independent experiments (B – D).

CI-994 and Ent consistently increased the expression of AKR1C1 protein to a greater extent than the other four remaining HDAC inhibitors in MCF7-AREc32 cells. Moreover, this activity is not peculiar to this cell line as both compounds also elevated AKR protein amounts in the epidermoid carcinoma A-431 cell line (Fig. 5A). For this reason, we restricted subsequent more in-depth analyses to these two compounds. We first confirmed by NRF2 knock-down (Fig. 5A) that these chemicals increase the expression of *AKR* mRNA (Fig. 5C & D) and protein (Fig. 5E & F) levels via this transcription factor in MCF7-AREc32 cells. Notably, however, these experiments also revealed that Ent and CI-994 were both less reliant upon NRF2 for augmentation of *AKR* expression than SFN. In a similar vein, we also noticed that expression of the luciferase reporter in response to Ent and CI-994– but not SFN – was largely independent of NRF2, in contrast to the endogenous *AKR* genes (*c.f.* Fig. 5B with Fig. 5C & D). Indeed, Ent displayed no dependence upon NRF2 in this experimental setting.

### HDAC inhibitor cytotoxicity is NRF2-independent

**Figure 6:**
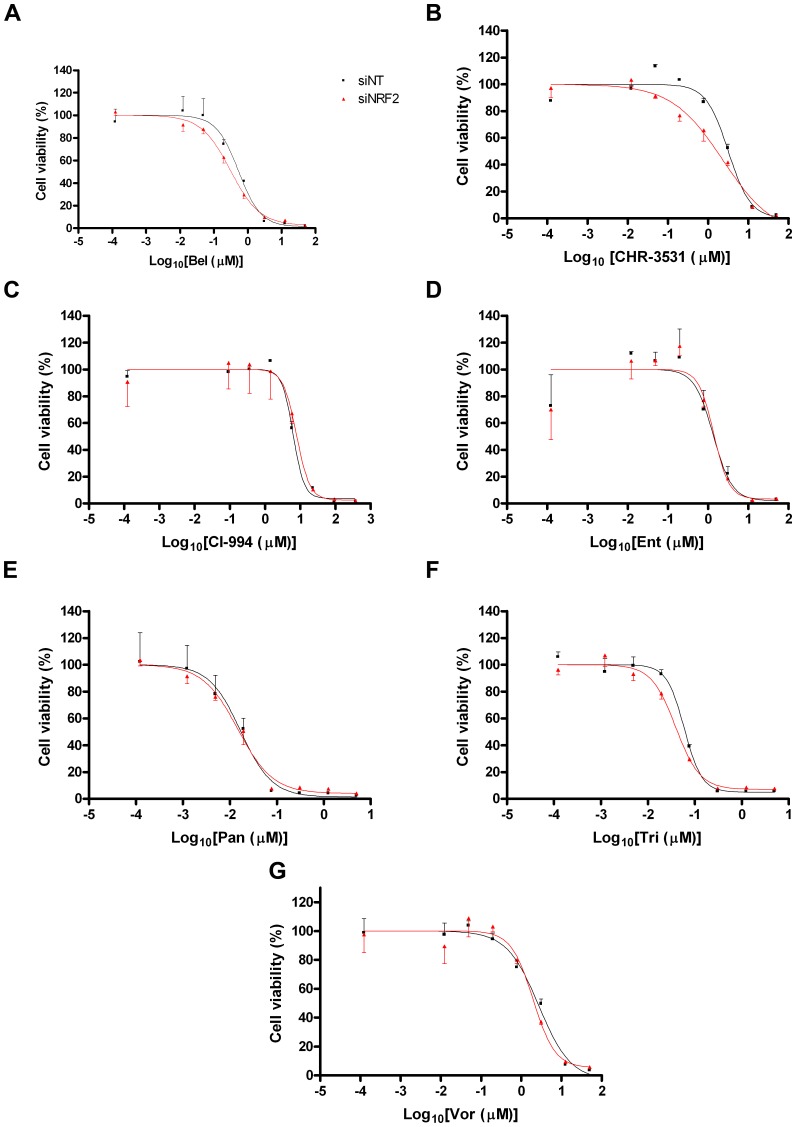
HDAC cytotoxicity is unaffected by NRF2 knock-down. MCF7-AREc32 cells were treated with non-targeting (siNT) or NRF2 targeting (siNRF2) siRNAs and, 48 h later, they were exposed to different doses of the indicated chemicals. Cell viability was assessed 72 h later. Data are presented as 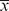 ±S.E.M of three independent experiments.

**Figure 7:**
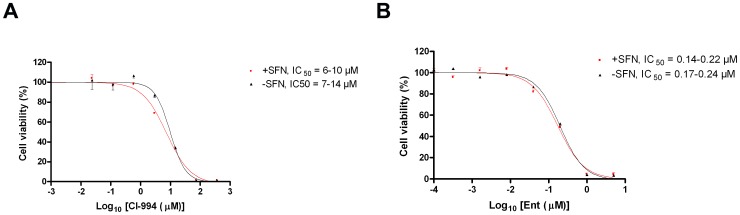
HDAC cytotoxicity is not diminished by prior activation of NRF2. MCF7-AREc32 cells were treated with 5 µM SFN or vehicle (DMSO). After 24 h had elapsed, they were exposed to various concentrations of CI-994 or Ent. Cell viability was assessed 72 h later. Data are presented as 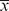 ±S.E.M of three independent experiments.

In the light of the finding that HDAC inhibitors increased NRF2 signalling and the expression of downstream genes, we tested whether NRF2 signalling affected the cytotoxicity of these compounds as single-use agents. In the first instance, we knocked-down NRF2 expression. At 48 h, at which point AKR expression is already significantly reduced, we exposed control or NRF2 knock-down cells to the various HDAC inhibitors. Cytotoxicity was assessed 72 h later. The data showed that the potency of these seven agents in this cell line is unaffected by inhibiting NRF2 signalling (Fig. 6). Secondly, we examined whether increasing NRF2 activity prior to exposure of cells to HDAC inhibitors might affect HDAC inhibitor efficacy. In these experiments, we primed cells with a low dose of SFN for 24 h before exposing the cells to the HDAC inhibitors. Again, no difference in Ent or CI-994 cytotoxicity was observed between the primed and the control cells (Fig. 7).

### Exposure of cells to HDAC inhibitors reduces the cytotoxicity of acrolein

**Figure 8:**
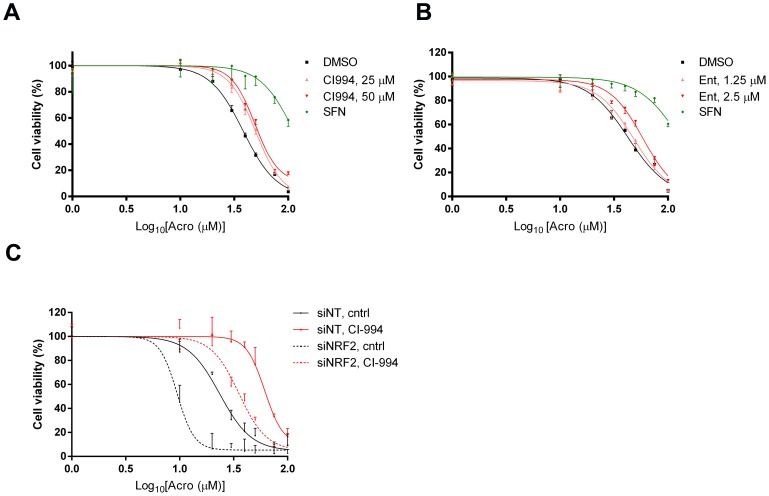
Pre-treatment with HDAC inhibitor CI-994 reduces the cytotoxicity of acrolein. **A & B**, MCF7-AREc32 cells were pre-treated with 5 µM SFN, the indicated doses of CI-994 and Ent, or vehicle (DMSO). After 24 h had elapsed, they were exposed to various concentrations of Acro. Cell viability was assessed 72 h later. Data are presented as 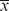 ±S.E.M of three independent experiments. **C,** MCF7-AREc32 cells were treated with non-targeting (siNT) or NRF2 targeting (siNRF2) siRNAs. The following day, they were pre-treated with 50 µM CI-994 or vehicle (DMSO). After 24 h of pre-treatment, cells were washed and exposed to different Acro. Cell viability was assessed 72 h later. Data are presented as 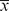 ±S.E.M of three independent experiments.

Although the cytotoxicities of the HDAC inhibitors themselves were not altered by diminishing or augmenting NRF2 signalling (Figs. 6 & 7), we investigated whether the ability of this class of inhibitors to activate NRF2 might blunt the toxicity of co-administered drugs. As a proof-of-principle, we examined acrolein (Acro), a lipid peroxide break-down product and a cytotoxic metabolite of cyclophosphamide. In agreement with earlier work [20], priming cells with SFN blunted the cytotoxicity of Acro (Fig. 8). Additionally, pre-treatment of cells with CI-994 increased the Relative IC_50_ value for Acro significantly (P<0.05 at both doses compared to control). For example, the Relative IC_50_ value for Acro in control cells was 36 µM versus 50 µM in cells pretreated with 50 µM CI-994. Ent also appeared to mitigate the cytotoxicity of Acro (Relative IC_50_ in control cells of 40 µM versus 65 µM in cells pre-treated with 2.5 µM Ent) but the effect was not statistically significant (Fig. 8). Finally, we confirmed that chemoprotection by CI-994 was partly NRF2-dependent by demonstrating that knock-down of the transcription factor exacerbated Acro toxicity in both control cells and cells pre-treated with CI-994 (P<0.05 in both populations). Unsurprisingly, CI-994 still offers some residual protection to NRF2 kd cells, consistent with our finding that it augments AKR protein levels to some extent even in the absence of the transcription factor (Fig. 5).

## Discussion

We have found that approximately 10% of a randomly chosen collection of largely clinical or pre-clinical drugs alter NRF2 signaling in the MCF7-AREc32 reporter cell line. These included the classic cytotoxic agents carmustine, lomustine, melphalan, in addition to the DNA intercalating agent epirubicin. Surprisingly, however, many of these drugs are targeted agents that lack the reactive moieties that might be expected to disrupt the NRF2-KEAP1 negative-feedback loop directly and, thus, stimulate NRF2 activity. These targeted drugs included an RXR agonist (bexarotene), an IGF1-R inhibitor (NVP-AEW541), a PIM-1 kinase inhibitor (Pim1 inhibitor 2), a PLK1 inhibitor (BI 2536) and – most strikingly – seven of nine tested HDAC inhibitors. These effects may be either indirect or due to the modulation of pathways directly involved in the regulation of NRF2 function.

For certain of the drugs tested, there is a mechanistic rationale why they influence NRF2 signalling. In the case of bexarotene, an RXR agonist, for example, the finding that this compound reduced NRF2 signaling (Fig. S3) is consistent with our earlier finding that RXR acts as a negative regulator of NRF2-ARE signalling [21]. This provides further evidence supporting the interaction. In other cases, it is more likely that the effects are indirect. For example, we found the IGF-1R inhibitor NVP-AEW541 stimulated NRF2 signalling, whereas a previous study reported that IGF-1 deficiency *in* *vivo* decreased NRF2 activity [22]. This difference could be explained if NVP-AEW541 has off-target effects. This is supported by the observation that the effects of NVP-AEW541 on ARE-driven gene expression were only partially NRF2-dependent (Fig. 2C).

The ability of HDAC inhibitors to activate NRF2 signalling was particularly striking. The fact that multiple, structurally dissimilar, HDAC inhibitors were identified in our screen suggests that this represents an on-target effect. NRF2 is known to be acetylated in a manner which influences its activity [23]. The other alternative is that HDAC inhibitors exert their effects by affecting chromatin organisation at NRF2-target genes. Two observations suggest that modifications to chromatin structure are important. Firstly, CI-994 and Ent had more profound effects upon expression of *AKR* genes than did the remaining HDAC inhibitors; these two inhibitors are differentiated from the others by their specificity [24]. They primarily target HDAC I and III proteins that specifically deacetylate histones. The remaining inhibitors are less specific and also inhibit HDACs that deacetylate non-histone proteins. Secondly, we consistently noted that HDAC inhibitors retained some ability to induce *AKR* genes even when NRF2 expression was suppressed. This can be rationalised if these inhibitors open up the chromatin structure and, thus, reduce the stringency of the requirement for NRF2 to stimulate *AKR* gene expression.

Our primary aim in performing this work was to examine whether drugs might disturb NRF2 signalling. Unanticipated NRF2 stimulation is clinically undesirable, having potentially deleterious consequences for drug efficacy and patient outcome. For example, NRF2 is a proto-oncogene and, therefore, drugs that inadvertently stimulate its activity might promote the survival of pre-existing pre-malignant cells [25]. For example, NRF2 activity promoted tumourigenesis in a genetically-engineered mouse model of pancreatic cancer, at least in part by mitigating oncogene-driven ROS formation [12]. Failure to control ROS also accounts for the finding that lack of NRF2 reduced tumour aggressiveness and increased survival in a *Braf^V600E^*-driven mouse model of lung cancer [26]. In addition to counteracting ROS, NRF2 may also support tumour formation by redirecting glucose and glutamine into anabolic pathways [11]. Finally, NRF2 signalling is undesirable because it drives a transcriptional response that confers a chemo-resistant phenotype on cells. By boosting chemo-resistance of tumours, targeted drugs that augment NRF2 activity might impair the cytotoxicity of co-administered cytotoxic agents to the detriment of patients. This is particularly the case as there is increasing interest in the use of targeted drugs, such as HDAC inhibitors, in combination with standard chemotherapies [24]. The potential for adverse drug-drug interactions between HDAC inhibitors and co-administered cytotoxic chemicals is exemplified by our observation that pre-treatment of MCF7-AREc32 cells with CI-994 impaired the cytotoxic effects of Acro, a reactive chemical and cyclophosphamide breakdown product. Although the magnitude of the drug-drug interaction observed between CI-994 and Acro was modest, many cytotoxic drugs have very narrow therapeutic windows and even a 50% increase in Relative IC_50_ values, such as observed here, might have adverse clinical consequences [27]. In addition, the efficacy of at least some stand-alone agents might be reduced by their own ability to stimulate NRF2 signaling. This is suggested by the observation that the cytotoxicity of NVP-AEW541 in MCF7-AREc32 cells is exacerbated by knocking-down NRF2 levels (Fig. 2D).

In summary, many anti-tumour agents have the unanticipated ability to stimulate NRF2 signaling. As a consequence, the effectiveness of certain therapies could be attenuated. It would therefore be appropriate to test for such effects as part of the drug development process.

## Supporting Information

Figure S1
**Chemotherapeutic alkylating agents stimulate NRF2 activity. Cell viability and luciferase activities were measured in separate plates of MCF7-AREc32 cells exposed to the indicated doses of the specified alkylating agents. Each measured parameter is plotted as 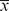 ±S.E.M of three independent experiments.**
(TIF)

Figure S2
**Stimulation of NRF2 activity by an IGF1-R inhibitor and a PIM-1 Kinase inhibitor. Cell viability and luciferase activities were measured in separate plates of MCF7-AREc32 cells exposed to the indicated doses of NVP-AEW541 or Pim1 Inhibitor 2. Each measured parameter is plotted as 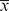
** ±**S.E.M of three independent experiments.**
(TIF)

Figure S3
**Epirubicin (Epi) and bexarotene (Bex) inhibit NRF2 activity. Cell viability and luciferase activities were measured in separate plates of MCF7-AREc32 cells exposed to the indicated doses of Epi or Bex. Each measured parameter is plotted as 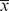
** ±**S.E.M of three independent experiments.**
(TIF)

Figure S4 **NRF2 knock-down in MCF7-AREc32 cells.** MCF7-AREc32 cells were treated with non-targeting (siNT) or NRF2 targeting (siNRF2) siRNAs. After 72 h had elapsed, total RNA was prepared, and the amount of *NRF2* mRNA determined by real-time quantitative PCR (**A**). Alternatively, cells were treated with SFN or vehicle (DMSO) for a further 2 h before preparing cell lysates and blotting for the indicated proteins (**B**). The band representing NRF2 is indicated with an asterisk.
(TIF)

Table S1 **Chemicals screened during this study. This table contains a complete list of the chemicals screened in the MCF7-AREc32 cell line during this study. The table also includes other relevant information regarding the compounds, including their supplier, presumed biological target, and clinical status.**
(XLSX)
