# Supplementary figures and images for: HDAC Inhibitors Increase NRF2-Signaling in Tumour Cells and Blunt the Efficacy of Co-Adminstered Cytotoxic Agents
Source: PLoS One. 2014 Nov 26;9(11):e114055. doi: 10.1371/journal.pone.0114055 (PMC4245243; doi:10.1371/journal.pone.0114055)

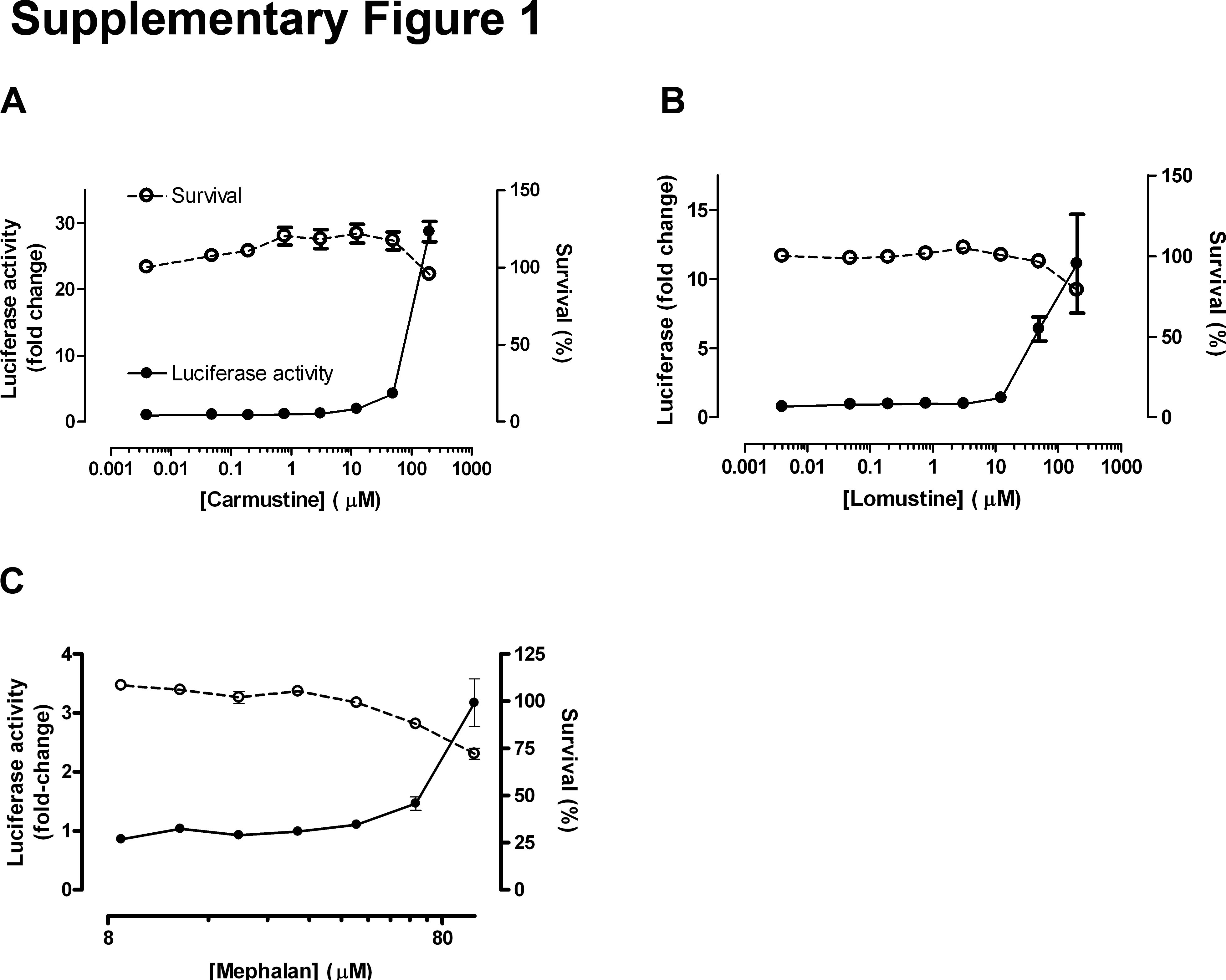

Supplement: Figure S1 — Chemotherapeutic alkylating agents stimulate NRF2 activity. Cell viability and luciferase activities were measured in separate plates of MCF7-AREc32 cells exposed to the indicated doses of the specified alkylating agents. Each measured parameter is plotted as ±S.E.M of three independent experiments. (TIF) [file pone.0114055.s001.tif]

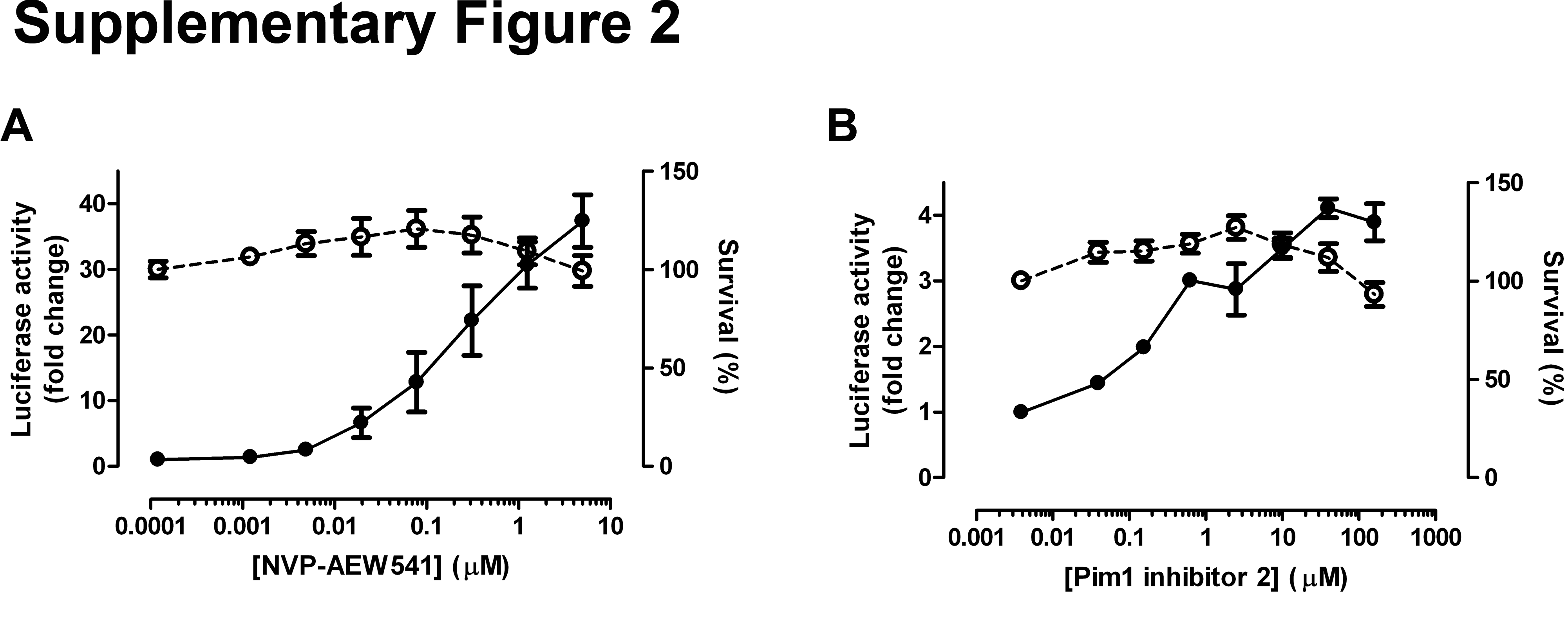

Supplement: Figure S2 — Stimulation of NRF2 activity by an IGF1-R inhibitor and a PIM-1 Kinase inhibitor. Cell viability and luciferase activities were measured in separate plates of MCF7-AREc32 cells exposed to the indicated doses of NVP-AEW541 or Pim1 Inhibitor 2. Each measured parameter is plotted as ±S.E.M of three independent experiments. (TIF) [file pone.0114055.s002.tif]

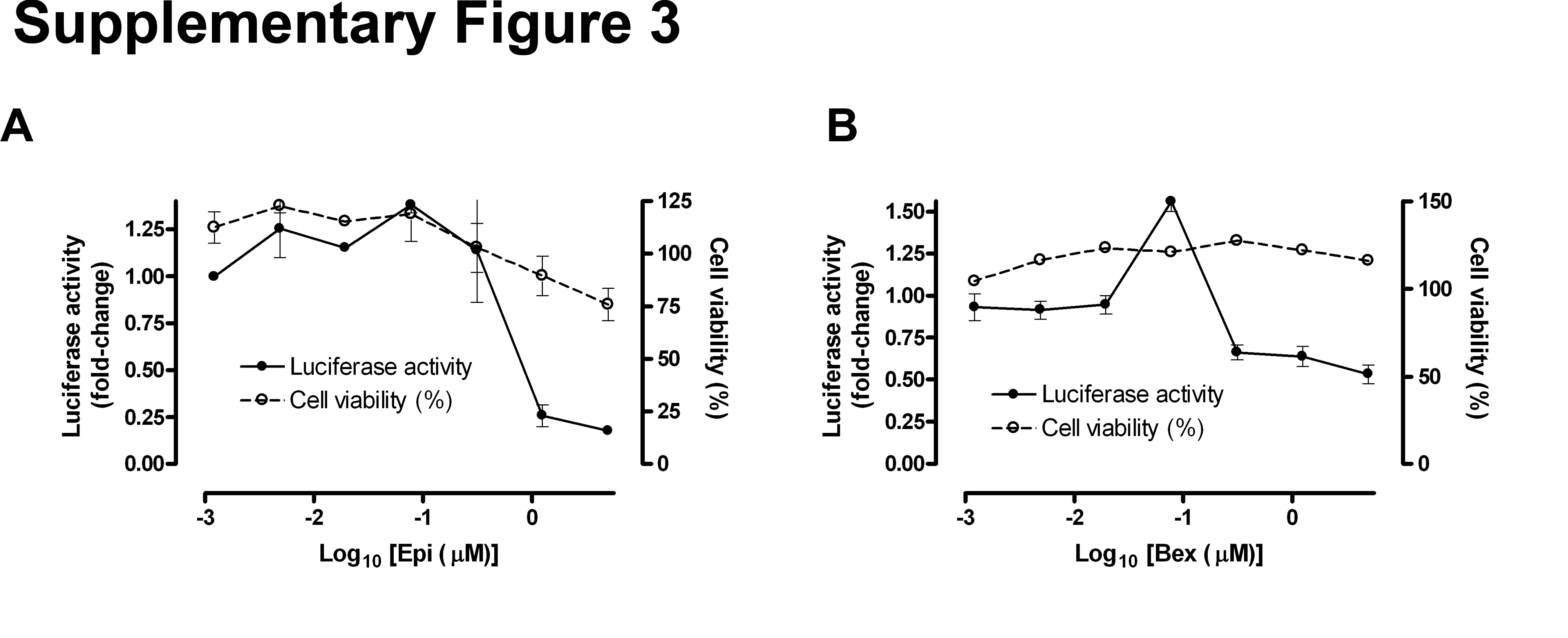

Supplement: Figure S3 — Epirubicin (Epi) and bexarotene (Bex) inhibit NRF2 activity. Cell viability and luciferase activities were measured in separate plates of MCF7-AREc32 cells exposed to the indicated doses of Epi or Bex. Each measured parameter is plotted as ±S.E.M of three independent experiments. (TIF) [file pone.0114055.s003.tif]

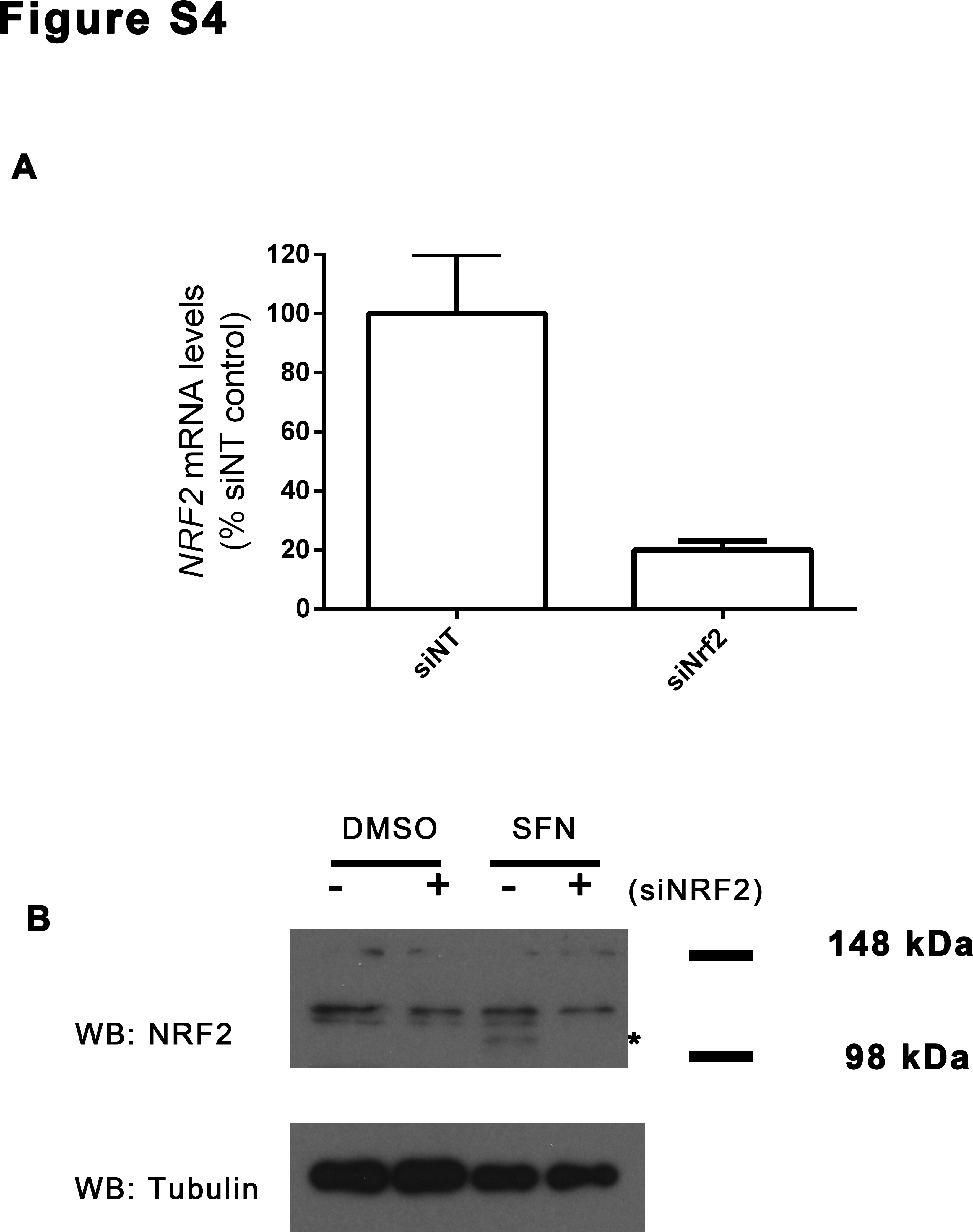

Supplement: Figure S4 — NRF2 knock-down in MCF7-AREc32 cells. MCF7-AREc32 cells were treated with non-targeting (siNT) or NRF2 targeting (siNRF2) siRNAs. After 72 h had elapsed, total RNA was prepared, and the amount of NRF2 mRNA determined by real-time quantitative PCR (A). Alternatively, cells were treated with SFN or vehicle (DMSO) for a further 2 h before preparing cell lysates and blotting for the indicated proteins (B). The band representing NRF2 is indicated with an asterisk. (TIF) [file pone.0114055.s004.tif]
